# An Energy-Efficient Flexible Multi-Modal Wireless Sweat Sensing System Based on Laser Induced Graphene

**DOI:** 10.3390/s23104818

**Published:** 2023-05-17

**Authors:** Jiuqing Feng, Yizhou Jiang, Kai Wang, Jianzheng Li, Jialong Zhang, Mi Tian, Guoping Chen, Laigui Hu, Yiqiang Zhan, Yajie Qin

**Affiliations:** 1 School of Information Science and Technology, Fudan University, Shanghai 200433, China; jqfeng20@fudan.edu.cn (J.F.); yzjiang18@fudan.edu.cn (Y.J.); kaiwang20@fudan.edu.cn (K.W.); lijz20@fudan.edu.cn (J.L.); jlzhang20@fudan.edu.cn (J.Z.); laiguihu@fudan.edu.cn (L.H.); yqzhan@fudan.edu.cn (Y.Z.); 2 Huashan Hospital, Shanghai 200040, China; mtian19@fudan.edu.cn

**Keywords:** laser-induced graphene, lactate enzyme electrode, wearable sensor, sweat sensing system, human motion monitoring, single-walled carbon nanotubes, health monitoring

## Abstract

Real-time sweat monitoring is vital for athletes in order to reflect their physical conditions, quantify their exercise loads, and evaluate their training results. Therefore, a multi-modal sweat sensing system with a patch-relay-host topology was developed, which consisted of a wireless sensor patch, a wireless data relay, and a host controller. The wireless sensor patch can monitor the lactate, glucose, K^+^, and Na^+^ concentrations in real-time. The data is forwarded via a wireless data relay through Near Field Communication (NFC) and Bluetooth Low Energy (BLE) technology and it is finally available on the host controller. Meanwhile, existing enzyme sensors in sweat-based wearable sports monitoring systems have limited sensitivities. To improve their sensitivities, this paper proposes a dual enzyme sensing optimization strategy and demonstrates Laser-Induced Graphene (LIG)-based sweat sensors decorated with Single-Walled Carbon Nanotubes (SWCNT). Manufacturing an entire LIG array takes less than one minute and costs about 0.11 yuan in materials, making it suitable for mass production. The in vitro test result showed sensitivities of 0.53 μA/mM and 3.9 μA/mM for lactate and glucose sensing, and 32.5 mV/decade and 33.2 mV/decade for K^+^ and Na^+^ sensing, respectively. To demonstrate the ability to characterize personal physical fitness, an ex vivo sweat analysis test was also performed. Overall, the high-sensitivity lactate enzyme sensor based on SWCNT/LIG can meet the requirements of sweat-based wearable sports monitoring systems.

## 1. Introduction

**Figure 1:**
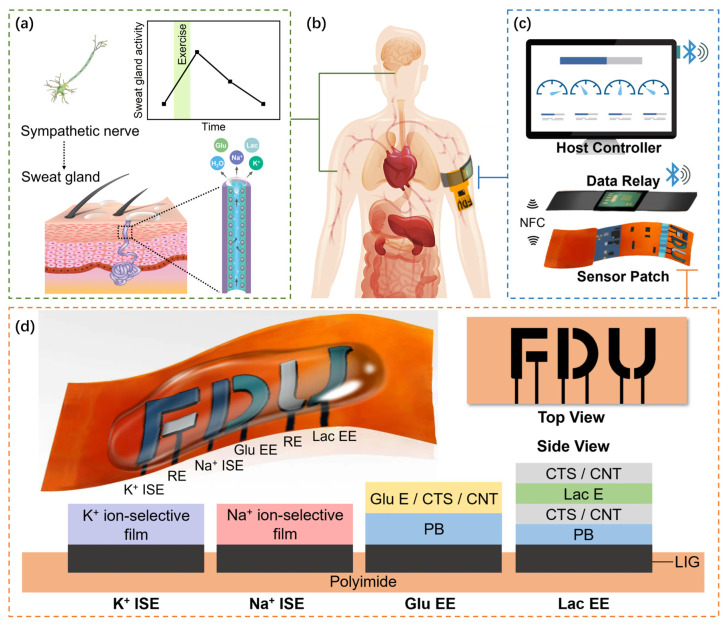
Application scenario of the multi-modal sweat sensing system: (**a**) the sympathetic nerve controls sweat gland activity levels in response to exercise; (**b**) the wireless sensor patch is stuck on human skin and the data relay can be fixed nearby to ensure enough NFC coupling; (**c**) the sweat sensing system consists of three parts: the wireless sensor patch, data relay, and host controller; (**d**) top view and side view of electrochemical sensors fabricated on a flexible Polyimide (PI) substrate.

With the development of digital sports, wearable sports monitoring devices have provided possibilities for scientific training and quantified the evaluation of athletes [1]. However, most commercial sports monitoring devices are limited to physical sensing, such as sensing the heart rate (HR) and blood pressure (BP), ignoring the enormous biochemical information provided by biofluids [2,3]. Sweat is easier to obtain and more suitable for continuous non-invasive monitoring than other biofluids such as blood, saliva, tears, and urine. In addition, it is more available during exercise [4]. Therefore, sweat has become an appropriate choice for real-time monitoring of athletes’ physical status. During physical exercise, the sympathetic nerves become excited and release acetylcholine, stimulating the sweat glands. Then, the sweat glands’ activity and secretion increase, as shown in Figure 1a. Various substances are present in sweat, such as lactate, glucose, K^+^, Na^+^, etc. A high Na^+^ concentration in sweat indicates hyponatremia and dehydration [5]. Furthermore, K^+^ is also vital for the human body. A low K^+^ concentration in sweat indicates that the communication of electrical impulses between cells is impaired, which not only induces muscle cramps but may also lead to hypokalemia [6]. Meanwhile, in the energy supply process, the conversion process of glucose and lactate is involved [7]. Therefore, monitoring sweat components contributes to the evaluation of physical status, quantification of training loads, and formulation of recovery strategies. Among them, lactate monitoring is crucial for specific populations, especially athletes performing high-intensity exercise to improve endurance. Lactate is considered a link between aerobic and anaerobic metabolisms and may accumulate during intense exercise due to glycolysis [8,9]. Furthermore, researchers have shown that lactate concentration increases during muscle fatigue [10,11,12], soft tissue compression, injury, and disease [9,13]. Therefore, lactate level is a good indicator in assessing athletes’ physical condition.

Compared to traditional colorimetric and optical methods, the electrochemical method has demonstrated outstanding advantages in sweat sensing due to its high selectivity [14,15,16,17], fast response time, and easily wearable design. Most electrochemical lactate sensors suitable for weakly acidic sweat utilize enzymes for specific recognition and yield current-mode (I-mode) outputs, which are measured by the chronoamperometry (CA) method [18,19,20]. However, most of the above works use traditional metals as electrode materials, which have a smaller specific surface area and often need help to immobilize enzymes well, leading to significant limitations in the sensitivity of enzyme sensors. Note that the traditional metal electrodes here do not include metal nanoparticles with metal nanoparticles but only refer to metal disc electrodes such as gold disc electrodes, platinum disc electrodes, etc. In contrast, laser-induced graphene (LIG), a class of three-dimensional (3D) porous carbon nanomaterials, has shown excellent physical and chemical properties, such as high carrier mobility, high thermal conductivity, and large specific surface area. Due to its large specific surface area, porous LIG can immobilize various proteins such as enzymes, antibodies, and receptors, showing a potential application in biosensing [21]. Apart from these, the fabrication process of LIG is simple and time-saving, and it is also feasible to produce complex shapes. Taking advantage of LIG’s promising benefits, several electrochemical sensors and wearable devices have been reported for biomarker detection [22,23]. However, as an essential biomarker, no LIG-based lactate sensor has been reported (to the best of our knowledge). To enhance the contact between the enzyme and electrode surface, the most current LIG-based enzyme sensors adopt the mixed solution immobilization method in that the enzyme and the fixative are prepared in a mixed solution (~1 mL limited by the range of balances) in the production process. However, the mixed solution immobilization method will cause large amounts of waste. Therefore, there are still unresolved difficulties in fabricating LIG-based lactate sensors.

In addition to the electrochemical sensors discussed above, developing multi-modal sweat sensing systems incorporating wearable electronic modules is a current trend [18,20,24]. These electronic modules generate bias and excitations for the sensors and collect the sensors’ responses. Finally, they could transfer data to host devices, which are commonly smartphones or computers with wireless receivers, enabling the developed wearable electrochemical devices access to the Internet-of-the-Things (IoT). These systems successfully observed biomarker concentration changes in subjects while exercising. However, Bluetooth (BT) modules communicate with host devices, and batteries are required to power this, limiting flexibility and wearable comfort [18,24]. As mentioned above, Radio-Frequency Identification (RFID) and Near-Field Communication (NFC) techniques are introduced in sweat sensing systems to realize wireless power delivery [20,25]. Compared to prior works, these NFC-based systems have relatively smaller sizes, but their communication distances are minimal. Since the wireless readers have to be fixed close to the wearable sensor patch, the application scenarios are restricted. Therefore, designing a sweat-sensing system that can address the current issues in size and communication distance would be significant. Meanwhile, the number of biomarkers detected by the published LIG-based flexible sweat sensing system is still limited, which is insufficient as a comprehensive reflection of physical status.

In order to overcome the challenges mentioned above, this work proposes a novel sweat lactate enzyme sensor based on a Single-Walled Carbon Nanotube (SWCNT)/LIG with a sandwich structure. This design allows the sensing layer to carry more enzymes, reduces overpotential, enhances the charge transfer between the enzyme and electrodes, and improves the electrochemical current of the sensor. Therefore, the sensitivity of the lactate enzyme sensor would be significantly improved. On this basis, a multi-modal sweat sensing system (as shown in Figure 1b) with the patch-relay-host topology has been developed and tested, as displayed in Figure 1c. Meanwhile, the number of biomarkers analyzed has been expanded by integrating LIG lactate, glucose, K^+^, and Na^+^ sensors into a single sensor patch.

## 2. Materials and Methods

### 2.1. Chemicals and Materials

SWCNT, glucose oxidase enzyme, polyvinyl butyral (PVB), polyvinyl chloride (PVC), chitosan (CTS), methanol, NaCl, FeCl_3_, CaCl_2_, MgCl_2_, ZnCl_2_, KCl, K_3_Fe(CN)_6_, hydrochloric acid (100 mM HCl), valinomycin, sodium tetrakis [3,5-bis (trifluoromethyl) phenyl] borate (Na-TFPB), bis (2-ethylhexyl) sebacate (DOS), tetrahydrofuran, acetic acid solution (2%, *w*/*w*), L- lactate, and D- glucose were purchased from Aladdin Biochemical Technology Co., Ltd. (Shanghai, China). Lactate oxidase enzyme and sodium ionophore X were purchased from Macklin Biochemical Technology Co., Ltd. (Shanghai, China). Phosphate buffered solution (10× PBS, containing 137 mM NaCl, 102.7 mM KCl, 8.1 mM Na_2_HPO_4_, and 1.8 mM KH_2_PO_4_) was purchased from Pinggen Instrument Co., Ltd. (Jiangxi, China). All reagents were analytical grade and used without further purification. The polyimide (PI) film (0.15 mm thin) was purchased from Chenxi Electronic Materials Co., Ltd. (Guangdong, China). The water-soluble tape was purchased from Yongri Co., Ltd. (Shanghai, China). The Ag/AgCl ink (JLL-20) was purchased from Julong Technology Co., Ltd. (Shanghai, China).

The laser engraving machine (CMA0604-B-A) was purchased from Han’s Yueming Co., Ltd. (Guangdong, China). The Scanning Electron Microscope (SEM) (proX) was purchased from Phenom Scientific Instrument Co., Ltd. (Shanghai, China). The stylus surface profiler (D-500) was purchased from KLA Co., Ltd. (San Francisco, USA). The confocal Raman system (Alpha300 R) was purchased from WITec Co., Ltd. (Ulm, Germany). The X-ray Photoelectron Spectroscopy (XPS) (pHI5300) was purchased from PerkinElmer Co., Ltd. (Waltham, MA, USA). The contact-angle system (OCA20) was purchased from Dataphysics Co., Ltd. (Stuttgart, Germany). The electrochemical analyzer (CHI600E) was purchased from CH Instruments. Co., Ltd. (Shanghai, China). The Stabilized Temperature Platform (STP) (P-20) was purchased from Jinglianghe Technology Co., Ltd. (Shenzhen, China). The magnetic stirrer (LC-DMS-H) was purchased from Lichen Technology Co., Ltd. (Hunan, China). The four-point probe system (FA1004) was purchased from Xingyun Electronic Equipment Co., Ltd. (Jiangsu, China). The ultrasonic cleaner (BK-360J) was purchased from Olabo Technology Co., Ltd. (Jinan, China). The dynamic signal analyzer (35670A) and power analyzer (N6705C) were purchased from Keysight Technologies Co., Ltd. (Santa Rosa, CA, USA).

The Field Programmable Gate Array (FPGA) (ICE5LP4K) was purchased from Lattice Semiconductor Co., Ltd. (Portland, OR, USA). The Successive-Approximation-Register (SAR) Analog-to-Digital Converter (ADC) (AD7091R) and the operational amplifiers (AD8657) were purchased from Analog Devices, Inc. (ADI) Co., Ltd. (Norwood, MA, USA). The instrumental amplifier (INA333) was purchased from Texas Instrument (TI) Co., Ltd. (Dallas, TX, USA). The NFC SoC (PN7462) was purchased from NXP Semiconductors Co., Ltd. (Eindhoven, The Netherlands). The BT Low Energy (BLE) SoC (nRF52840) and the BLE USB-dongle (nRF52832) were purchased from Nordic Semiconductor Co., Ltd. (Trondheim, Norway).

### 2.2. Dual Lactate Enzyme Sensing Optimization Strategy

LIG is a relatively novel material that can be generated on a PI substrate through laser induction. Due to its excellent characteristics, it can be widely used in electrochemical sensors. However, no reports on lactate enzyme sensors based on LIG electrodes have been reported. The improved fixing of enzymes on the electrode surface is the key to fabricating a lactate enzyme sensor. In this way, a stable working environment can be created for the enzyme protein molecule on the working electrode surface, which enables the enzyme to maintain its structure, mobility, and biocatalytic activity as much as possible as well as interact directly or indirectly with the electrode through an electronic transfer medium [26]. Therefore, improving enzyme sensors’ performance usually involves optimizing electrode material modification and enzyme immobilization. This paper proposed the dual lactate enzyme sensing optimization strategy as follows:

1. Electrode material optimization strategy

The electrode material optimization strategy can be implemented from two directions: changing the electrode material itself and decorating the electrode with some material. Traditional metal materials have a small specific surface area and cannot immobilize protein molecules well, significantly limiting the sensitivity of enzyme sensors. Therefore, LIG with a large specific surface area can theoretically improve the sensitivity of enzyme sensors. Apart from this, since size effects, surface effects, quantum size effects, and macroscopic quantum tunneling effects decorate nanomaterials on the electrode, this can also increase the specific surface area of the sensor so that the sensing layer can carry more enzymes. In addition, this can reduce overpotential and improve the oxidation-reduction current of biological molecules, further enhancing the charge transfer between enzymes and electrodes [27]. Commonly used nanomaterials include Carbon Nanotubes (CNTs), gold nanoparticles (AuNPs), and platinum nanoparticles (PtNPs). In this work, SWCNT nanomaterials were used for decoration.

2. Enzyme immobilization optimization strategy

The material used for immobilizing enzymes must have good biocompatibility and not cause enzyme inactivation. CTS, as a cheap and easy-to-obtain material, is often used to enhance the immobilization of enzymes and electrodes because its chemical structure contains many N and O functional groups that serve as covalent modification starting points [28], and the material is easy to obtain and cheap. Therefore, this paper used CTS as the adhesive for enzymes.

### 2.3. Fabrication of LIG Electrodes

The flexible sensor array consists of Na^+^ and K^+^ sensors, glucose (Glu) and lactate (Lac) enzyme sensors, as well as reference electrodes (REs), as displayed in Figure 1d. First, a flexible PI film was cut into the appropriate size for sensor substrates. Then, a piece of PI film was taped onto a glass slide with water-soluble tape and placed into a laser engraving machine. Next, graphene was induced with optimal parameters, such as laser power and scan speed (details given in Section 4). The pattern of letters F, D, and U was designed with AutoCAD (Autodesk). LIG samples with an average sheet resistance (Rs) of 8.71 Ω/square were used to fabricate electrochemical electrodes. It takes less than 1 min to fabricate a complete LIG electrode array.

### 2.4. Fabrication of Ag/AgCl Reference Electrodes

Ag/AgCl ink was uniformly coated on the LIG electrode pattern and cured at 100 °C for 45 min. For the Voltage-mode (V-mode) detection, NaCl (≥1.4%, *w*/*w*) and PVB (10%, *w*/*w*) were dissolved in methanol, ultrasonic stirred for 30 min, and dropped in 0.5 μL and 1.4 μL reference solutions on two electrodes, respectively, and dried at −4 °C in the dark [18]. However, for the I-mode detection, the Ag/AgCl reference electrode has not been modified by NaCl and PVB. During the sweat analysis, Ag/AgCl reference electrodes were also used as the counter electrodes to simplify the sensing system.

### 2.5. Fabrication of Enzyme Sensors

Prussian blue (PB) can promote the generation of the sensing current and increase the sensitivity of I-mode sensors. In this work, PB thin films were prepared by chemical synthesis. Due to the flat surface of traditional metal electrodes, an additional adhesive such as poly (diallyl dimethyl ammonium chloride) (PDDA) and CTS are often used to enhance connectivity between the PB and electrodes. However, some adhesives may affect the sensor’s performance due to poor conductivity. In contrast, the LIG used in this study had a porous structure that allows for a strong connection between the LIG electrode and PB through capillary action without adhesives.

The CTS solution (1%, *w*/*w*) was first prepared by dissolving CTS in an acetic acid solution and magnetically stirring for 1 h. Then, SWCNT (2 mg/mL) was added to the CTS solution by ultrasonic agitation for over 30 min to prepare a viscous solution (CTS/CNT), which was essential to enhance the connectivity of the oxidase enzyme with the electrode surface. Before enzyme immobilization, 3 μL of fresh PB solution (containing 2.5 mM FeCl_3_, 100 mM KCl, 2.5 mM K_3_Fe(CN)_6_, and 100 mM HCl) was drop-casted on LIG electrodes to deposit the PB mediator layer [18].

For the lactate enzyme sensors, the traditional mixed solution method is inappropriate due to the cost and instability of the lactate enzyme. As such, a sandwich structure was proposed (as Figure 1d) to reduce waste. First, cover a layer of viscous solution to enhance connectivity. Then, drip-coat the lactate enzyme on the viscous layer, and finally, cover a layer of viscous solution to wrap the lactate enzyme in it, similar to the sandwich structure. An amount of 3 μL of the CTS/CNT solution was drop-casted onto the PB/LIG electrode; the electrode was later covered with 3 μL of lactate enzyme solution (5 × 10^3^ unit/μL in PBS of pH 5.0), and finally, 3 μL of the CTS/CNT solution [18].

For the glucose enzyme sensors, a glucose enzyme solution (1 unit/μL in PBS of pH 5.0) was mixed thoroughly with the CTS/CNT viscous solution at the volume ratio of 2:1. Then, drop-cast 3 μL of Glu E/CTS/CNT solution onto the PB/LIG electrode [18]. The enzyme sensors and all solutions were stored at −4 °C when not in use.

### 2.6. Fabrication of Ion-Selective Sensors

To prepare the K^+^ selective solution, valinomycin (2%, *w*/*w*), Na-TFPB (0.6%, *w*/*w*), PVC (32.7%, *w*/*w*), and DOS (64.7%, *w*/*w*) were dissolved in tetrahydrofuran and ultrasonic stirred for 30 min. To prepare the Na+ selective solution, Sodium ionophore X (1%, *w*/*w*), Na-TFPB (0.6%, *w*/*w*), PVC (33%, *w*/*w*), and DOS (65.4%, *w*/*w*) were dissolved in tetrahydrofuran and ultrasonic stirred for 30 min. Then, 4μL of K^+^ selective solution and 10 μL of Na^+^ selective solution were dropped on the LIG electrodes’ surface, respectively, and then dried naturally at −4 °C [18].

## 3. System Design and Implementation

**Figure 2:**
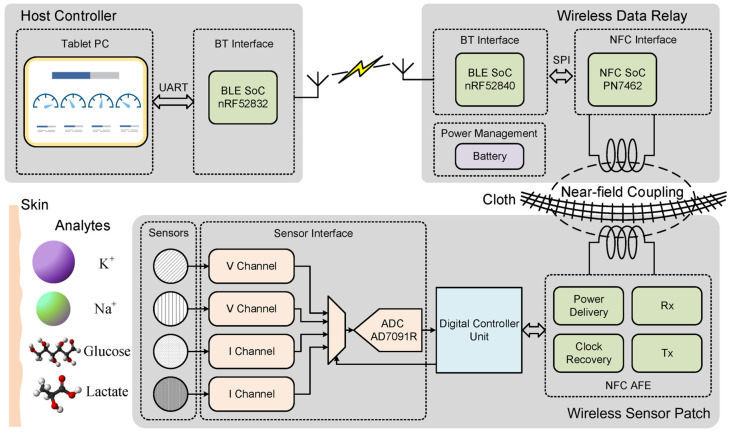
Block diagram of the proposed multi-modal wireless sweat sensing system.

Figure 2 draws the block diagram of the multi-modal sweat sensing system with the proposed patch-relay-host topology. The wireless sensor patch is stuck on human skin, such as the upper arm, collects response from flexible LIG electrochemical electrodes, and sends digitalized data to the host controller via a wireless data relay. The wireless data relay is fixed on a cuff near the wireless sensor patch, powers the wireless sensor patch by NFC, and realizes data interchange simultaneously. Finally, the host controller visualizes the received data.

### 3.1. Wireless Sensor Patch

**Figure 3:**
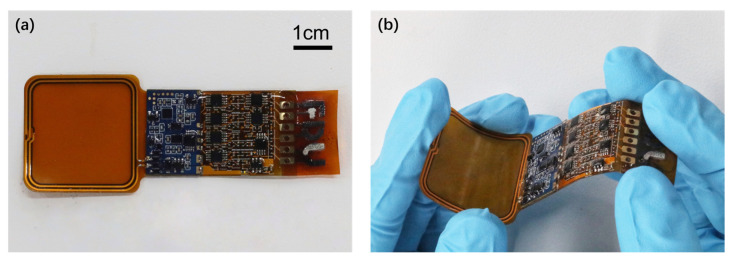
Photograph of the wireless sensor patch. (**a**) Top view and (**b**) bending.

The wireless sensor patch is made up of electrochemical LIG sensors, sensor interface circuitry, a customized digital control unit (DCU) implemented in an FPGA, the NFC analog front end (AFE), and some auxiliary circuits, as shown in Figure 3a,b. To improve the wearability, the circuit part of the wireless sensor patch is built on a flexible printed circuit (FPC), where the FPGA and some core circuits are packaged on a conventional rigid printed circuit board (PCB) due to their smaller footprints and higher density.

#### 3.1.1. Electrochemical LIG Sensors

Four types of electrochemical LIG sensors are implemented on a single LIG electrode array, including a lactate enzyme sensor, glucose enzyme sensor, Na^+^ ion-selective sensor, and K^+^ ion-selective sensor (details given in Section 2). Electronic responses generated by the four sensors enter the sensing channels in parallel. The electronic responses of lactate and glucose sensors are in the I-mode, while the Na^+^ and K^+^ sensors are in the voltage-mode (V-mode).

#### 3.1.2. Sensor Interface

A four-to-one analog multiplexer connects two V-mode and two I-mode sensing channels to a 12-bit SAR ADC. All the channels can be selected individually by generating different control signals to the multiplexer. Figure S1 gives the schematics of the voltage and current sensing channels. The V-mode input is amplified by an instrumental amplifier and filtered by a fourth-order Sallen–Key low-pass filter. In contrast, the I-mode input is first amplified by a trans-impedance amplifier (based on the operational amplifier). As the central controller, The DCU generates control signals and reads ADC results.

#### 3.1.3. DCU

Reducing the time the RF field is turned on is the key to increasing the system’s energy efficiency. Therefore, the wireless sensor patch involves a customized DCU (displayed in Figure S2). The customized DCU controls data collection and NFC communication as the core part. It runs the control logic when the wireless sensor patch is powered on. It generates the selected signals to the sensor interface, enables the ADC socket to collect data, and controls the NFC digital baseband to receive commands and send results. Its time decomposition of every work step after power-on is shown in Figure S3.

In addition, since it controls the operation of all the modules, the design of the DCU adopted many low-power techniques. The wireless sensor patch involves three operating states, i.e., IDLE, READY, and ACTIVE. The ADC socket and most function modules in the NFC digital baseband are powered off in the IDLE state to save energy. After receiving a valid NFC request, it turns into the READY state for anti-collision. Once the communication is established, all the modules are enabled in the ACTIVE state. At the same time, gray code and ripple counter are utilized to reduce power consumption.

#### 3.1.4. NFC AFE

The NFC AFE (shown in Figure S4 and Table S1) comprises four parts: power delivery circuits, clock recovery circuits, Rx circuits, and Tx circuits. Once the wireless data relay turns on the RF field, the 13.56 MHz sinusoidal carrier passes through the bridge rectifier and low dropout regulator (LDO) to generate the 3.3 V power supply. It also enters an inverter to output a 13.56 MHz digital clock for the DCU. In addition, the Rx circuit and Tx circuit are used to realize the functions of load modulation and envelop demodulation.

### 3.2. Wireless Data Relay

**Figure 4:**
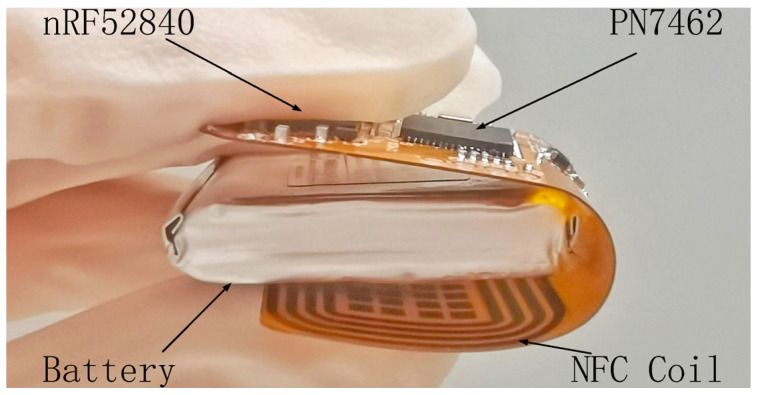
Photograph of the wireless data relay.

The portable wireless data relay shown in Figure 4 is designed as a folding structure with an NFC interface and a BT interface connected by an SPI bus. The battery-powered data relay is proposed to improve the comfort of the wearable wireless sensor patch and avoid the risk of battery leakage damage to human tissues. Utilizing an NFC SoC operating under the ISO14443-A communication protocol, the NFC interface generates an RF field to power the wireless sensor patch and transfers digitalized data from the sensor to the BT interface. Moreover, the application of the BT improves the communication distance, enabling convenient data transfer to the host controller. The BT interface, implemented with a BLE SoC operating in slave mode, forwards commands from the host controller to the NFC interface and transfers data from the NFC interface to the host controller. The wireless data relay is designed to be battery-powered so it can be conveniently carried and communicated with the wireless sensor patch. To reduce power consumption, the RF field of the NFC interface can be turned off when the NFC communication is not active.

### 3.3. Host Controllers

**Figure 5:**
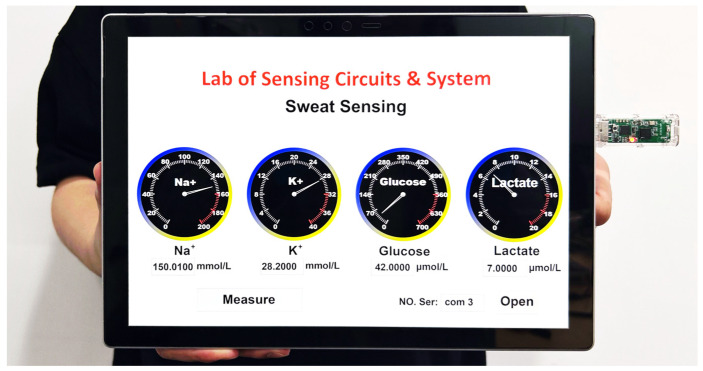
The host controller notes that the GUI is running on a tablet PC.

A tablet Personal Computer (PC) connected with a BLE USB dongle operates as the host controller, as shown in Figure 5. The USB dongle connects with the wireless data relay in the BLE master mode. It receives data and sends commands to the wireless sensor patch via the relay. A Graphical User Interface (GUI) developed with Python and PyQt is utilized to visualize the data. Users could also send commands to the system by clicking buttons on the GUI. Moreover, the host controller can be calibrated before each use to eliminate variation.

## 4. Results

### 4.1. Morphological and Physical Characterization

**Figure 6:**
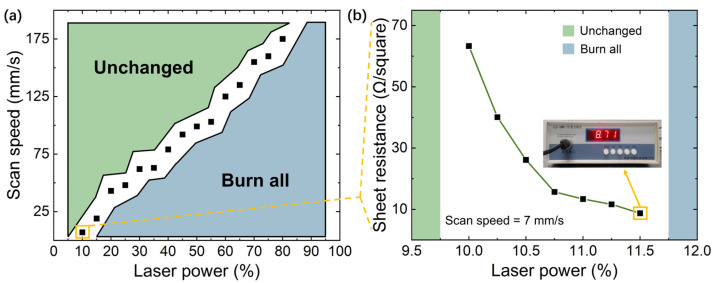
Selection of laser inducement parameters (laser power and scan speed). (**a**) The relationship between laser power and scan speed to induce LIG. In the green region, no obvious carbonization occurred. In the blue region, the PI material was burned. When the parameters are in the white space, LIG could be achieved; (**b**) sheet resistance measurement for laser power fine-tuning.

For the fabrication of LIG, laser power and scan speed are the two most important parameters. To find the optimal parameters, the above two parameters are dynamically changed (Figure 6a). In order to minimize the processing deviation each time, 7 mm/s was selected as the scan speed. In addition, 9.5~12% of the maximum power (80 W) was selected for fine-tuning around 10% of the maximum power (Figure 6b). When the laser power is 11.50% of the maximum power, the sheet resistance is the least (8.71 Ω/square), which is the optimal power for LIG.

**Figure 7:**
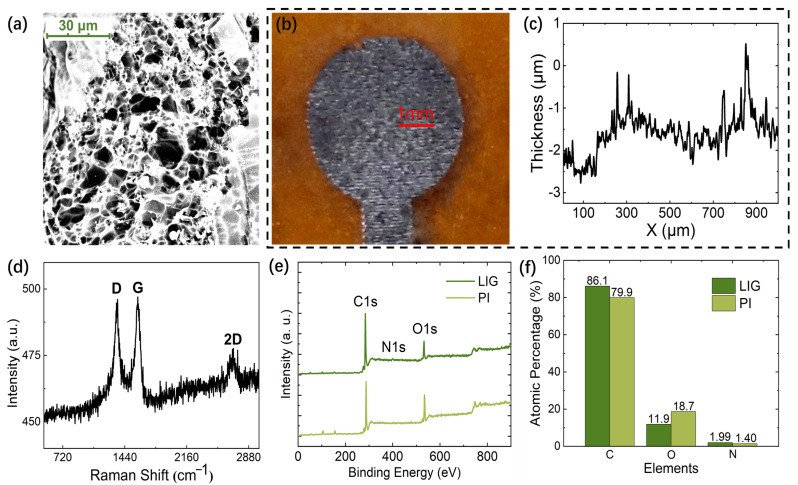
(**a**) SEM image of pristine LIG. (**b**) Sample scanned by stylus surface profiler. (**c**) Surface roughness of the LIG. (**d**) Raman spectrum of the LIG demonstrates prominent D, G, and 2D peaks that suggest a significant degree of graphene formation during the inducement process on the polyimide. (**e**) XPS full spectrum of LIG and PI. (**f**) XPS element spectrum of LIG and PI.

We monitor the LIG morphology induced with optimal parameters. In detail, the LIG morphology was first characterized by an SEM operating at 10 kV acceleration voltage. The SEM image (Figure 7a) shows the nanoscale filaments overlapped with LIG’s loose and porous morphology. This unique structure provides a high surface area for following sensing material immobilization. The surface roughness of the LIG was acquired using a stylus surface profiler. By horizontally scanning a 1 mm sample (Figure 7b), a very rough and disordered LIG surface with micrometer-high peaks and valleys was observed in Figure 7c, which resulted in the increment of the specific surface area.

In addition, we also employed Raman spectroscopy further to characterize the composition and structural properties of the LIG. The Raman spectra were obtained through a confocal Raman system with 532 nm laser wavelength and 1800 lines/mm grating. An objective lens of 100× magnification and 0.95 numerical aperture (NA) was used, and the spot size was about 500 nm in diameter. The Raman spectrum (Figure 7d) shows several typical bands (D, G, and 2D) of graphene according to previous reports [22,29]. The sharp G band (1350 cm^−1^) and 2D-band (2700 cm^−1^) demonstrate the presence of the graphene crystalline structure in fabricated LIG. Notably, the D-band has been demonstrated to be associated with the disorder of the graphene lattice; thus, the prominent D-band located at ~ 1350 cm^−1^ in this work suggests that LIG has many defects, such as the five- and seven-carbon atom rings [30]. Meanwhile, the ratio of the D- and G-bands also effectively indicate the defect concentration in graphene. The obtained result of I(D)/I(G) = 1.00 indicates that the defects in the LIG possess a relatively high concentration.

To further investigate the surface elemental composition of LIG, XPS was used for characterization. The full spectrum of XPS in Figure 7e shows a C1s main peak at 284.5 eV, corresponding to sp^2^ hybridized carbon atoms in the LIG. An O1s peak at 533.1 eV and an N1s peak at 400.1 eV was also observed. The elemental composition of LIG and PI is shown in the XPS elemental spectrum in Figure 7f, in which the content of C, O, and N in the LIG is 86.1%, 11.9%, and 1.99%, respectively. In contrast, the C, O, and N content in the PI is 79.9%, 18.7%, and 1.40%, respectively. Compared to the PI substrate, LIG exhibits higher C content and lower O content, indicating that the PI was graphitized after laser induction. In particular, LIG contains 1.99% of the N element due to the incomplete removal of the N element in PI during the laser induction process.

The above characterizations demonstrate the excellent properties of LIG materials for electrochemical sensors. However, the practicability of LIG-based sensors for wearable applications still requires further discussion.

**Figure 8:**
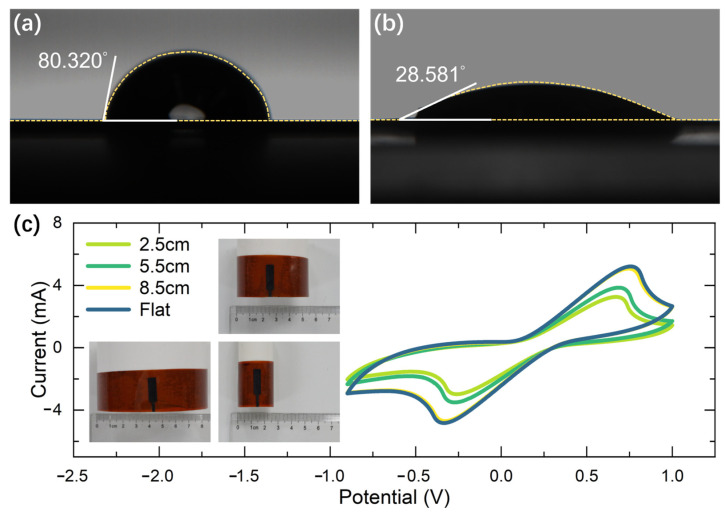
(**a**) Contact angle measurement of the PI substrate. (**b**) Contact angle measurement of the LIG showing hydrophilic behavior. (**c**) The comparison of cyclic voltammogram under various bending statuses (redox species include 1 mM K_3_[Fe(CN)_6_] and 1 mM K_4_Fe(CN)_6_, the electrochemical probe is a bare LIG electrode, and the reference electrode is a calomelan electrode).

Contact angle measurements were conducted using a contact-angle system. A drop of deionized water (around 10 μL) was placed on the surface of interest and the contact angle was calculated. The study of the wettability of PI and LIG present shows contact angles of φ = 80.320° and φ = 28.581° (Figure 8a,b), which highlights the hydrophilicity of LIG produced by laser-induced PI. The hydrophilic surface could be more helpful for sweat to come into contact with the LIG electrodes.

The mechanical stability of LIG-based sensors is still a concern, as the LIG flakes are prone to de-lamination over repeated bending or stretching. This limits their use in wearable applications where the sensors need to endure prolonged and repeated deformations. To demonstrate the mechanical stability of the flexible LIG-based sensor, cyclic voltammetry (CV) is characterized under various bending conditions [31,32,33,34]. The cylindrical rods used for the bending test and the radii of curvature were 4.25 cm, 2.75 cm, and 1.25 cm, respectively. Figure 8c compares CV under various bending statuses and the stabile redox current. It can be seen that the LIG electrodes under different curvature radii all have pronounced oxidation peaks and reduction peaks. It can be inferred that the bending structures with a flat or 8.5 radius show no apparent difference in redox peaks. However, as the radius increases, the reduction peaks move in the positive direction while the oxidation peaks move in the negative direction.

### 4.2. Electrochemical Characterization of the Sensors

**Figure 9:**
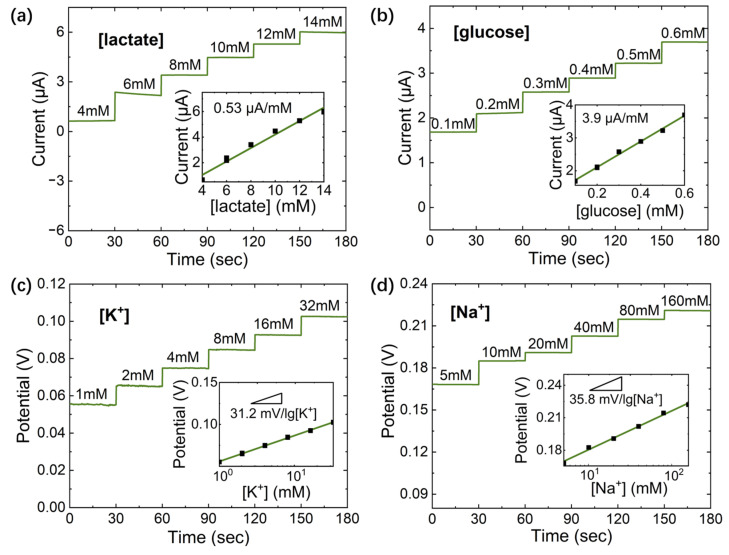
The CA responses and sensitivity of (**a**) lactate enzyme sensors (applied potential is 0 v) and (**b**) glucose enzyme sensors (applied potential is 0 v) and the open circuit potential responses and sensitivities of (**c**) K^+^ ion-selective sensors and (**d**) Na^+^ ion-selective sensors.

The electrochemical response of ion-selective and enzyme sensors was measured by chronopotentiometry (CP) and CA methods, respectively, using an electrochemical analyzer. The analyte concentrations used in the electrochemical characterization refer to the concentration range in real human sweat (K^+^: 1–32 mM; Na^+^: 5–160 mM; glucose: 0.1–0.6 mM; and lactate: 4–14 mM). Figure 9a–d show good linearity and obvious response value, which indicates that the proposed sensors are capable of human sweat monitoring. The measured sensitivities of lactate, glucose, K^+^, and Na^+^ are 0.53 μA/mM, 3.9 μA/mM, 31.2 mV/decade, and 35.8 mV/decade, respectively.

**Figure 10:**
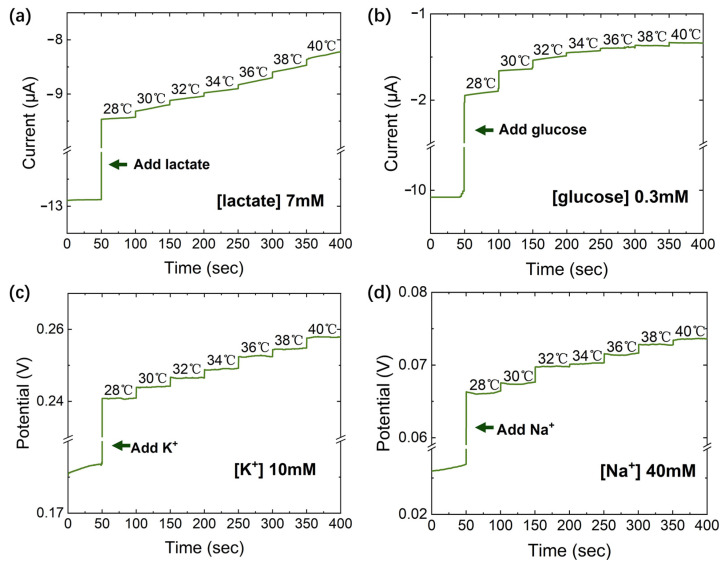
Temperature effect of sensors. (**a**) Lactate; (**b**) glucose; (**c**) K^+^; (**d**) Na^+^.

The selectivity of the ion-selective sensor was tested based on commercial Ag/AgCl reference electrodes. Figure S5a,b draw the potential response through the interfering ions (K^+^, Na^+^, Ca^2+^, Mg^2+^, and Zn^2+^) that were selected to refer to actual human sweat. In addition, the solution temperature was changed to the median of the standard concentration range of the human body’s lactate, glucose, K^+^, and Na^+^ in the human body (lactate: 7 mM, glucose: 0.3 mM, K^+^: 10 mM, and Na^+^: 40 mM). According to the Nernst equation, the potential of an ion-selective sensor gradually increases with increasing temperature, whereas, according to the Michaelis–Menten equation, the reaction rate of enzyme-catalyzed reactions increases with an increase in temperature before reaching an optimum temperature. Therefore, it is essential to investigate the effect of temperature on various sensors. Figure 10a–d show that in the range of 28~40 °C, the current and voltage rise slightly but the effect of the temperature change is less than the minimum detectable concentration on the voltage/current. Furthermore, after 15 days stored at −4 °C, the sensitivity decreases of the glucose and lactate sensors are within 10%, and that of the K^+^ and Na^+^ ion-selective sensors are less than 5% (as shown in Figure S6).

### 4.3. Portable and Wearable Devices Characterization

In order to verify the performance of the sensor interface designed in this paper, the gain-frequency responses of sensing channels were tested utilizing a dynamic signal analyzer. As shown in Figure S7a, the gain of the current channel was 507.6 kΩ, and the low-pass cut-off frequency was 1.017 Hz. Both test results tolerate the range of the sensors’ response and fit the design well. Figure S7b shows that the gain of the voltage channel is 8.9 dB (2.79 V/V), and the low-pass cut-off frequency was 0.82 Hz.

For wearable applications, power consumption is critical. The total power consumption of the wireless data relay and wireless sensor patch was measured with a power analyzer. The power analyzer was used to supply 3.7 V to the wireless data relay that powers the wireless sensor patch via the NFC. The measured current waveform is drawn in Figure S8. The consumed supply current is 11 mA when the system is on standby. In addition, the BLE has to listen for commands periodically, causing small spikes in the waveform. Moreover, the RF waveform of the data collection–transmission phase is drawn in Figure S9. The typical data-collecting task is shorter than 200 ms, and the devices consume an extra 67.8 mJ per data-collection task compared to the standby condition, which proves its low power consumption.

### 4.4. In Vitro Test of the System

**Figure 11:**
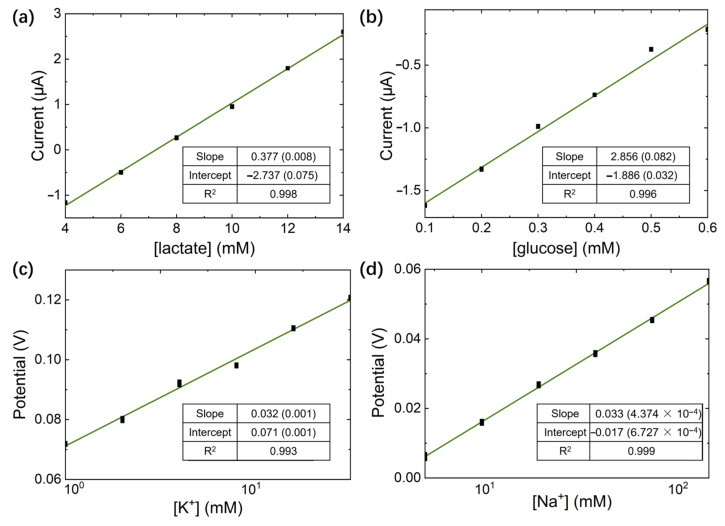
**The** in vitro test results of the lactate, glucose, K^+^, and Na^+^ sensors. (**a**) lactate enzyme sensors, (**b**) glucose enzyme sensors, (**c**) K^+^ ion-selective sensors, and (**d**) Na^+^ ion-selective sensors.

In an in vitro test of this system, solutions of the analyte were applied to the flexible sensor, which is connected to the circuit part, and the data were obtained from the GUI. The linear fitting relation of lactate, glucose, K^+^, and Na^+^ were 0.38 μA/mM (lactate), 2.86 μA/mM (glucose), 32.5 mV/1 g[K^+^], and 33.2 mV/1 g[Na^+^] (Figure 11), respectively. The system maintained a stable linear relationship between the electronic signals received from the host controller and the analyte concentration. Table 1 summarizes the performance of all the sensing functions that exhibited wide operating ranges and sufficient sensitivity.

### 4.5. Ex Vivo Test of the System

**Figure 12:**
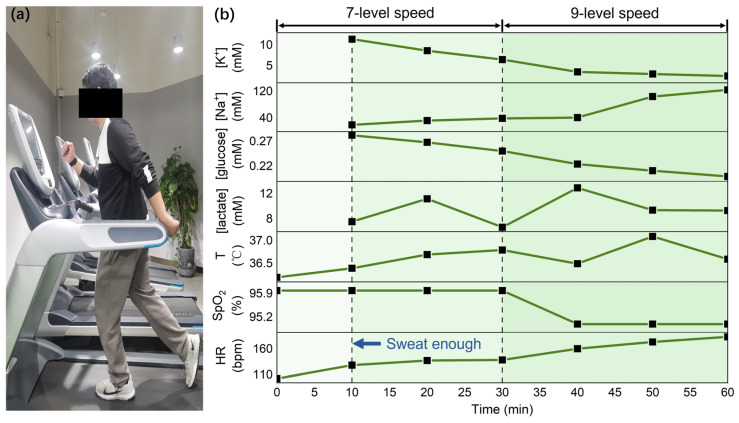
(**a**) A subject is running and (**b**) ex vivo sweat analysis during running (T = Temperature).

An ex vivo system performance evaluation was performed on a subject during running exercises, as displayed in Figure 12a. The protocol involved 30 min of running at a seven-level speed and 30 min of running at a nine-level speed (speed coefficient of the treadmill, ranging from 1 to 20). Sweat samples were collected every 9~10 min from the subject’s forehead and wiped after every collection [18]. The sweat is collected by scraping the forehead sweat with a scraper and putting it in a 1.5 mL centrifuge tube. Moreover, HR and oxygen saturation (SpO_2_) was measured by clamping an external commercial fingertip pulse oximeter on the finger, and body temperature was recorded by scanning the forehead with an external commercial forehead thermometer. All data were collected after sweat start (~15 μL/cm^2^), as shown in Figure 12b. The sweat start rate of 15 μL/cm^2^ was obtained by dividing the minimum sweat volume limited by the collection tool by the approximate forehead area for sweat collection. To make the experimental results more informative, biosensor responses as a function of HR during ex vivo sweat analysis are drawn in Figure S10. The placement and attachment of the components for the complete systems are shown in Figure S11.

The temperature rises and falls during the exercise process, which is the combined effect of exercise and sweat evaporation. The HR increases significantly when speed increases and remains relatively constant at each speed level. SpO_2_ decreased slightly throughout the exercise but remained within the normal range (>94%). To a certain extent, sweat glucose can represent blood glucose [35,36], which decreases concentration during exercise. Athletes are prone to hyponatremia because of the loss of Na^+^ through sweat during strenuous exercise [37]. Hence, Na^+^ is an important biochemical marker for judging electrolyte imbalance. K^+^ and Na^+^ are inflow/outflow through ion channels, and once the ion channel is activated, K^+^ flows out and Na^+^ flows in. Therefore, the concentration of K^+^ in sweat decreases, and Na^+^ increases. The lactate concentration builds up gradually during exercise but then declines as the athlete balances out. Meanwhile, as the speed level increases, the average value of SpO_2_ decreases while the average value of lactate concentration increases, further confirming that SpO_2_ and lactate concentration have an opposite trend.

## 5. Discussion

This work first proposed the SWCNT/LIG lactate sensor made by the dual enzyme sensing optimization strategy, which has a sandwich structure and exhibits excellent sensitivity. In addition, the simultaneous multi-modal sensing of lactate, glucose, K^+^, and Na^+^ is realized on a single patch. Compared to [18,38,39], the sensitivity of lactate in this work is 2.5 times, 59.0 times, and 7.6 times larger, respectively. Furthermore, the glucose sensitivity is at least 1.7 times larger than others (as shown in Table 2). Apart from this, compared to [18,24,25,38,39], this work intellectually introduced the low-power wireless data relay to realize the trade-off of wearability and communication distance. Due to the wireless power link, there are no batteries in the wearable sensor, removing the risk of leakage. Moreover, the proposed system could achieve a more extended communication distance by utilizing the wireless relay than those systems that only use NFC for communication.

## 6. Conclusions

In this paper, a multi-modal sweat sensing system based on patch-relay-host topology was developed to measure the four critical components of sweat, i.e., lactate, glucose, K^+^, and Na^+^, which can reflect one’s physical status during sports. Among them, the SWCNT/LIG lactate enzyme sensor was based on a dual lactate-enzyme sensing optimization strategy and was fabricated for the first time by sandwich-structured drop-casting. Due to the large specific surface area and capillary action of SWCNT/LIG, more enzymes can be carried, bringing the electrochemical lactate sensor to an improved sensitivity of 0.53 μA/mM. The wireless sensor patch was manufactured on flexible substrates, offering comfort for people wearing it. The wireless data relay placed on garments or in the cuff powers the sensor as well as transmits data and commands between the host controller and wireless sensor patch.

The characterizations and tests of the LIG material, sensors, sensing channels, and communication modules were given. In addition, the power consumption and data collection–transmission test imply the functionality of the sweat sensing system. Finally, the system’s ex vivo test displays its mature applications and performance and lays the foundation for in vivo experiments in the future. The system’s function could be further extended by introducing electrochemical sensors for other analytes. The signal acquisition modules may also be optimized by integrating the sensor interface and NFC interface into an SoC. At the same time, developing algorithms for a health assessment with sweat is also helpful.

## Figures and Tables

**Table 1:** Summary of sensing performance.

Sensing Function	Lactate	Glucose	K^+^	Na^+^
Gain of amplifier	510 kΩ	510 kΩ	3	3
Analyte concentrations	2.8 mM–15.8 mM	76 μM–1.8 mM	1 mM–32 mM	5 mM–160 mM
Sensitivity of sensors	0.53 μA/mM	3.9 μA/mM	31.2 mV/decade	35.8 mV/decade
Sensitivity of system	3.2 μM	0.42 μM	14 μM	53 μM
			(@ 1 mM)	(@ 10 mM)

**Table 2:** Comparison of performance between LIG-based electrochemical sensors and other sensors.

Items	[18]	[24]	[25]	[38]	[39]	This Work
Sensing mode	V, I	V	V, I	I	V, I	V, I
Electrochemical sensors	K^+^, Na^+^, lactate, glucose	K^+^, Na^+^, Cl^–^, pH	K^+^, Na^+^, pH, glucose	Lactate	Glucose, pH, lactate, Cl^−^, urea	K^+^, Na^+^, lactate, glucose
Material	Au	Au	AgNWs ^*^	Carbon ink	Au	LIG
Sensitivity of lactate	0.22 μA/mM	-	-	0.009 μA/mM	0.07 μA/mM	0.53 μA/mM
Sensitivity of glucose	2.35 μA/mM	-	0.75 μA/mM	-	1.3 μA/mM	3.9 μA/mM
Powering	Battery	Battery	NFC	Battery	NFC	NFC
Communication	Bluetooth	Bluetooth	NFC	Bluetooth	NFC	Bluetooth + NFC
Communication Distance	6–10 m	6–10 m	≤0.1 m	6–10 m	≤0.1 m	6–10 m
Texture	PCB	PCB	FPC	Polyurethane	PCB + FPC	PCB + FPC
Footprint	9 × 2.7 cm^2^	8.3 × 3.2 cm^2^	3.7 × 1.5 cm^2^	6.5 × 6.5 cm^2^	6.2 × 5.7 cm^2^	4.5 × 2 cm^2^

* AgNWs (Silver nanowires).

## Data Availability

The data presented in this study are available on request from the corresponding author.

## Supplementary Materials

The following supporting information can be downloaded at: https://www.mdpi.com/article/10.3390/s23104818/s1.
