# Supplementary material for: An Energy-Efficient Flexible Multi-Modal Wireless Sweat Sensing System Based on Laser Induced Graphene
Source: Sensors (Basel). 2023 May 17;23(10):4818. doi: 10.3390/s23104818 (PMC10221498; doi:10.3390/s23104818)
Supplement: Supplementary file 1 [file sensors-23-04818-s001.zip › sensors-2345925-supplementary.pdf]

An Energy-efficient Flexible Multi-modal Wireless Sweat Sensing System Based on Laser Induced Graphene

Supplementary Materials

Jiuqing Feng[1], Yizhou Jiang[1], Kai Wang[1], Jianzheng Li[1], Jialong Zhang[1], Mi Tian[2], Guoping Chen[1,*], Laigui Hu[1], Yiqiang Zhan[1] and Yajie Qin[1,*]

[1] School of Information Science and Technology, Fudan University, Shanghai, 200433, China

[2] Huashan Hospital, Shanghai 200040, China

*Corresponding author. Email: yajieqin@fudan.edu.cn (Y.Q.); gpchenapple@fudan.edu.cn (G.C.)

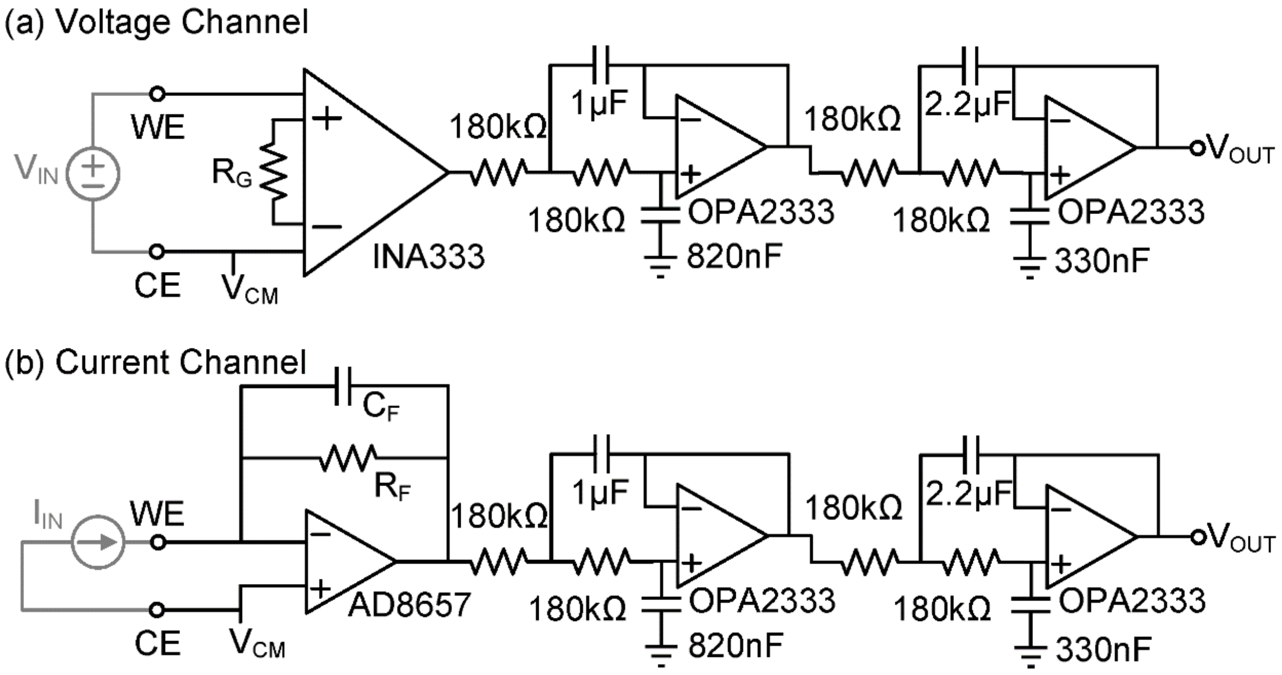

Figure S1: Schematic of the (a) voltage and (b) current sensing channels in the wireless sensor patch.

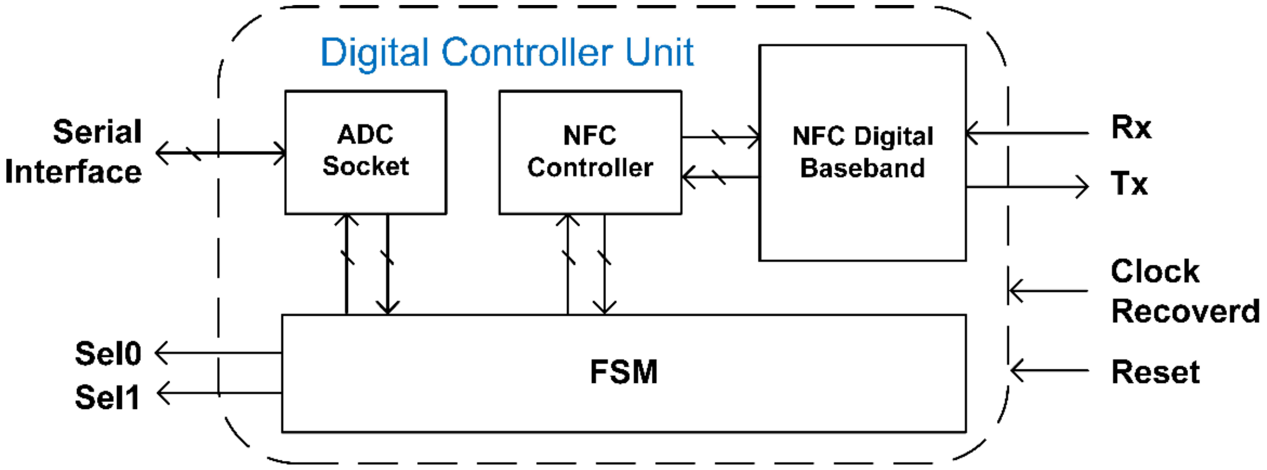

Figure S2: Block diagram of the DCU.

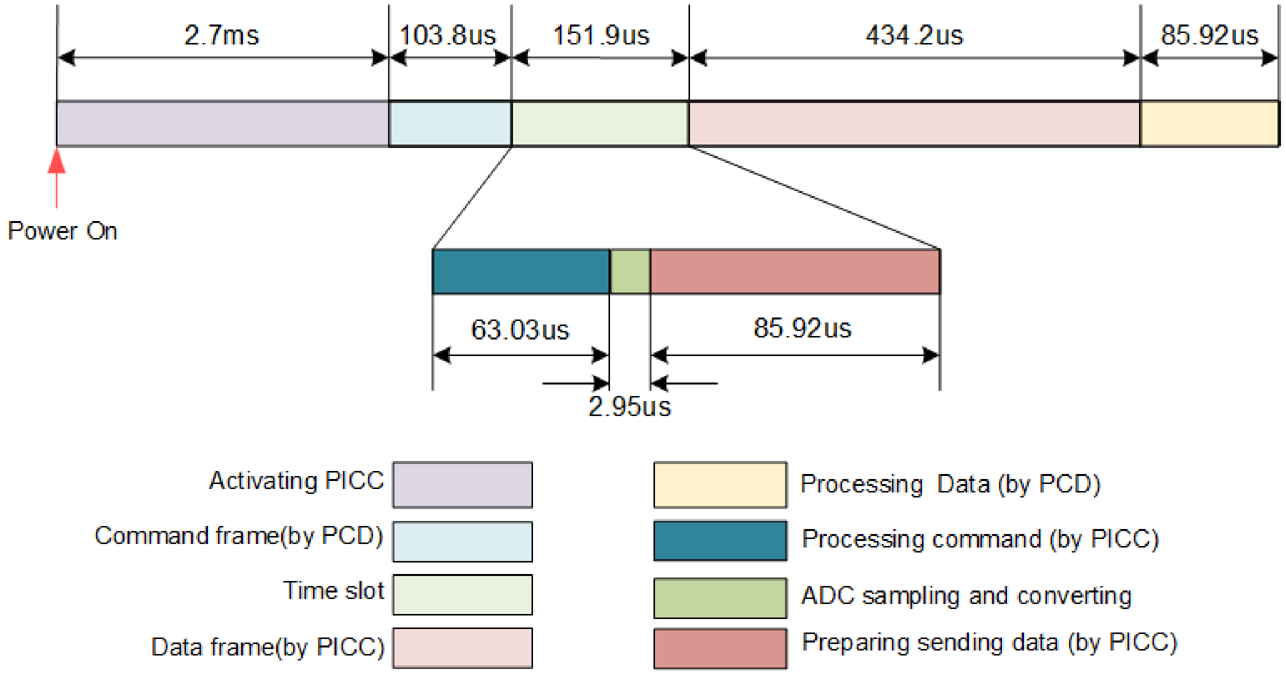

Figure S3: Timing diagram of the working process.

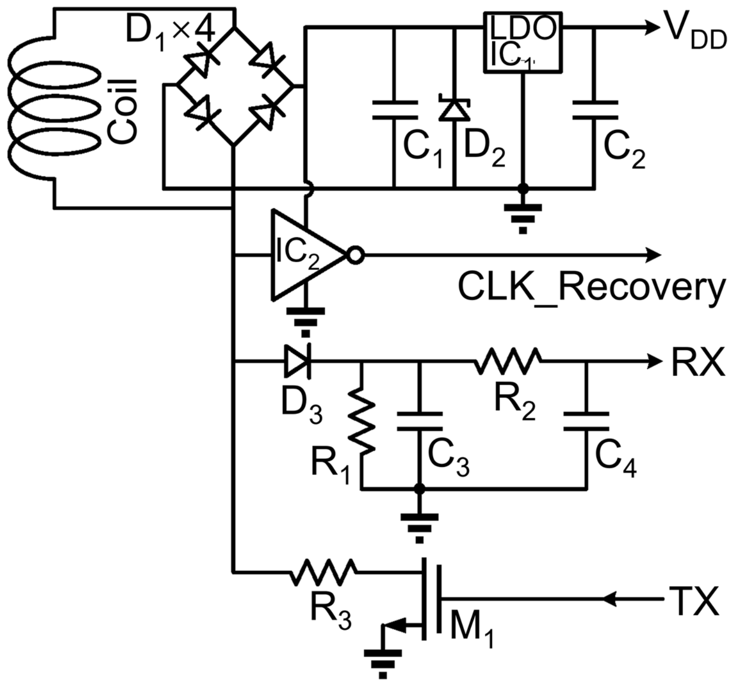

Figure S4: Schematic of the NFC AFE.

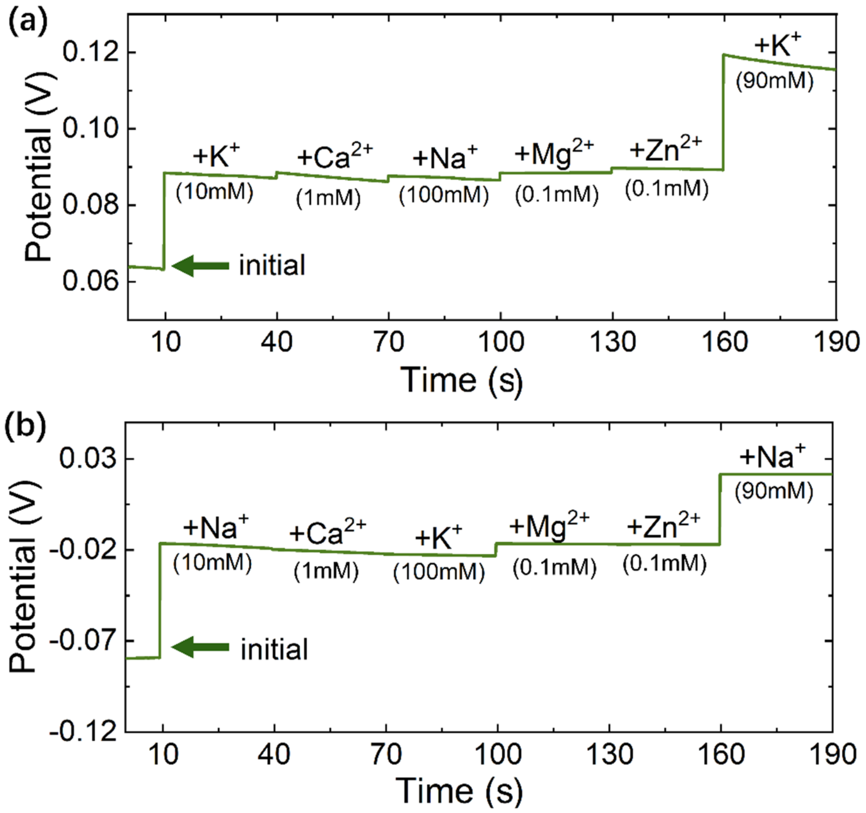

Figure S5: CP curve of ISEs electrode selectivity test results. (a) K^+ and (b) Na^+.

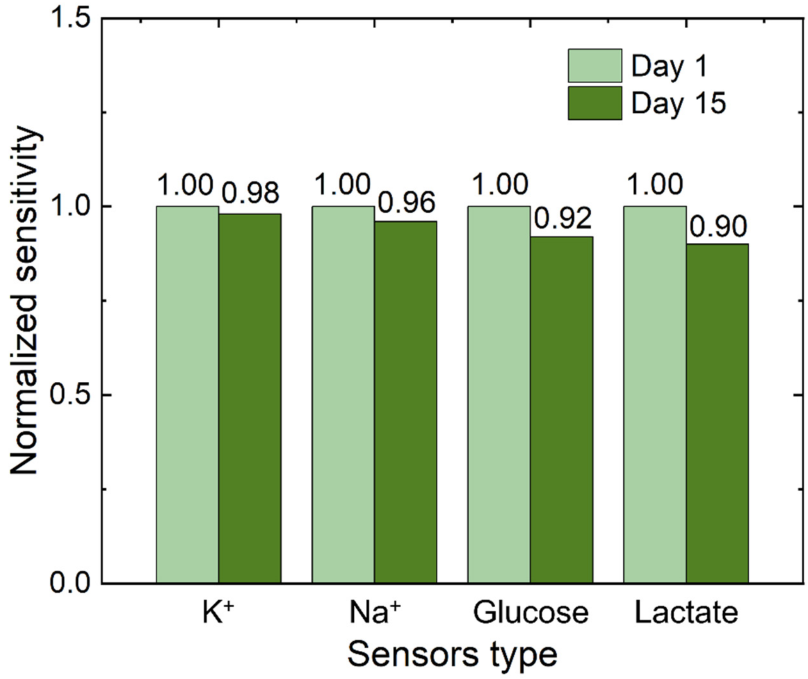

Figure S6: Long-term stability of the sensors.

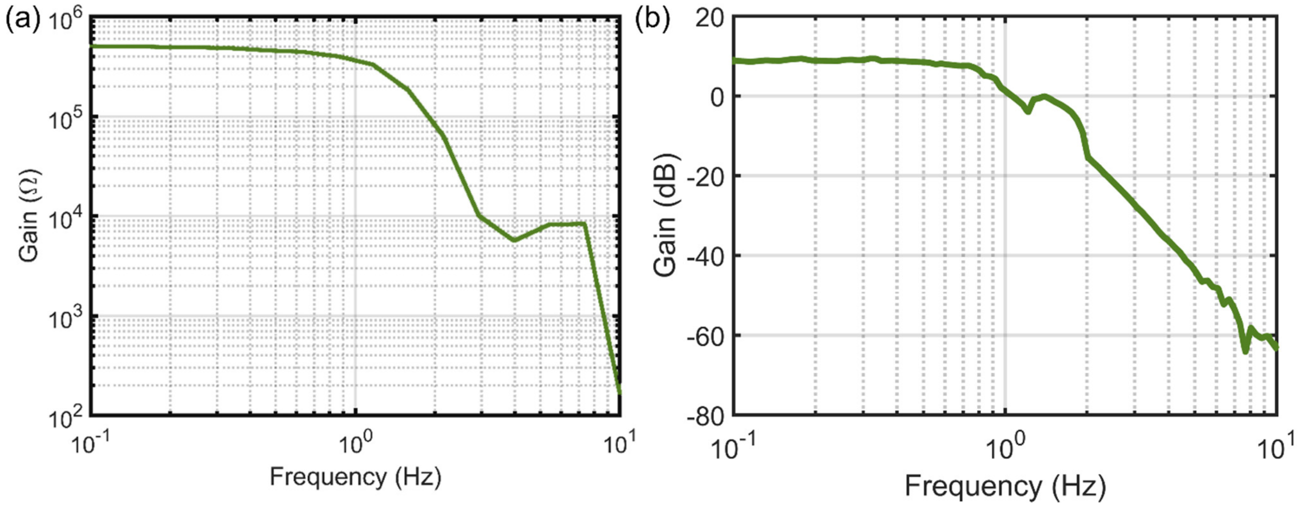

Figure S7: Measured gain-frequency response of the (a) I-mode channel (Transimpedance = 510 kΩ) and (b) V-mode sensing channel (Gain = 3 V/V).

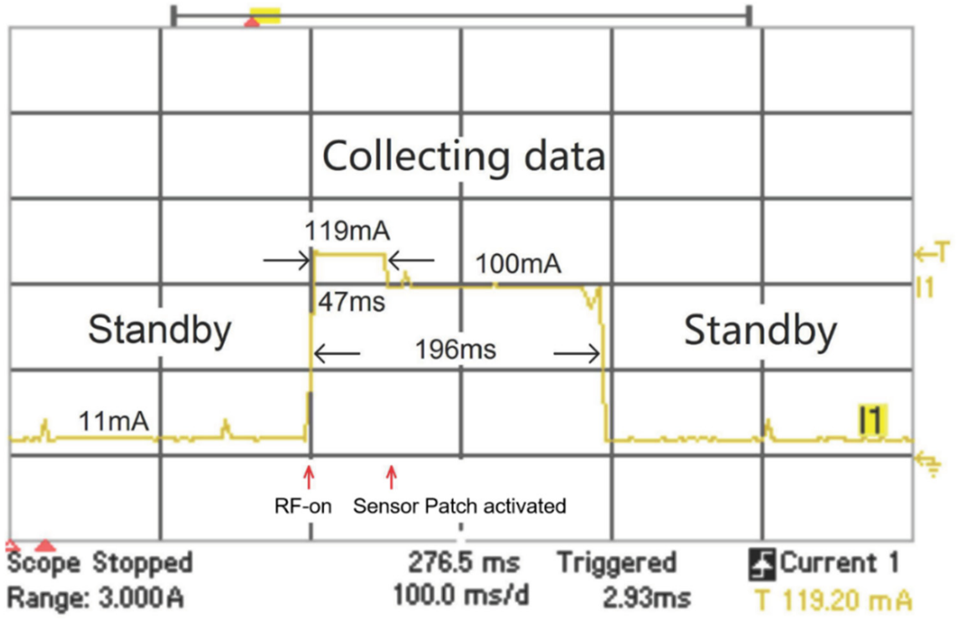

Figure S8: The current waveform of a typical sweat sensing task.

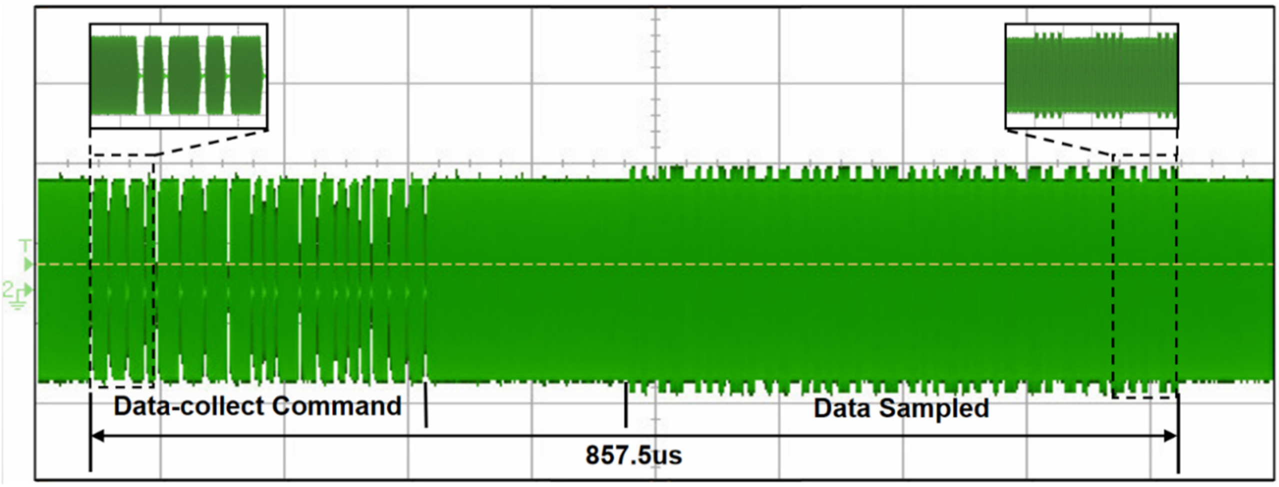

Figure S9: The RF waveform of a typical data collection and transmission task.

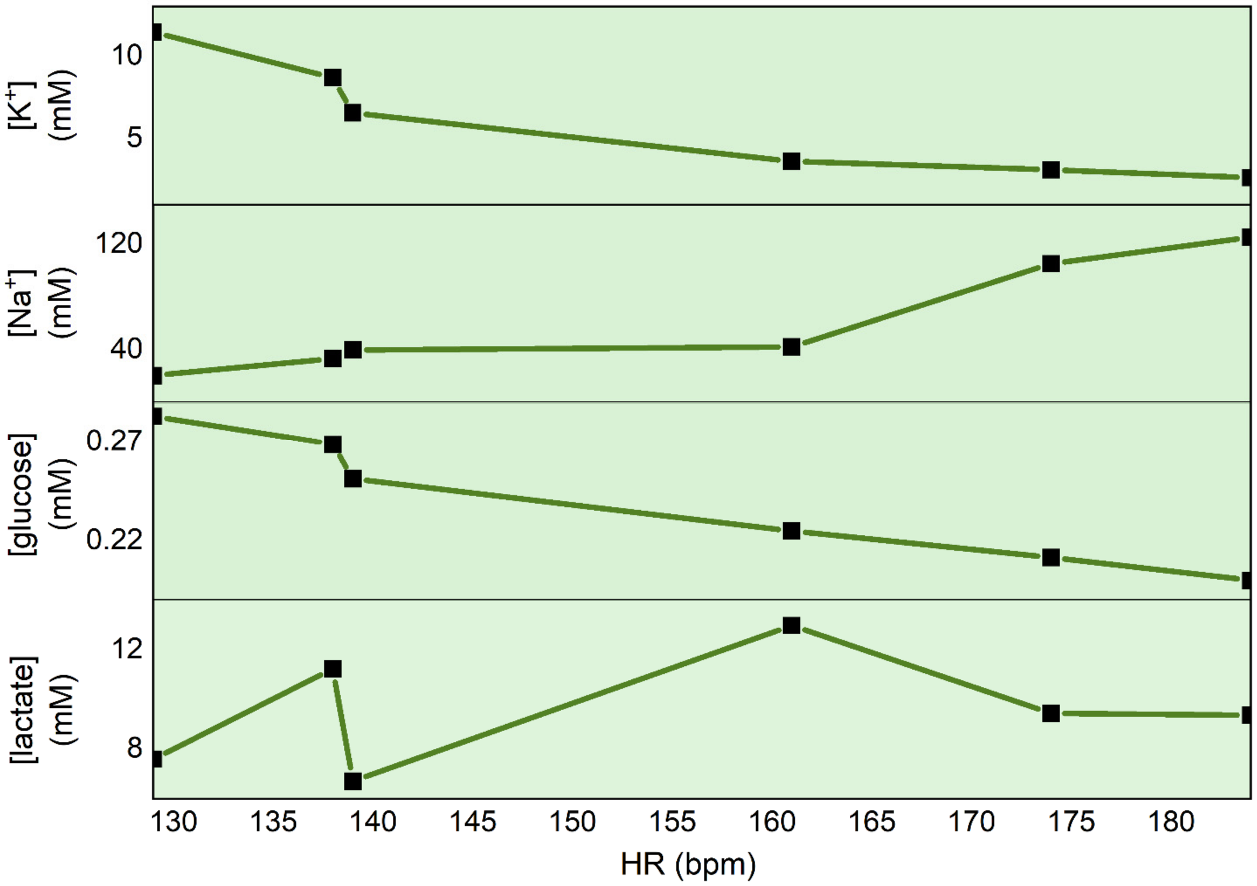

Figure S10: Biosensor responses as a function of HR *ex-vivo* sweat analysis.

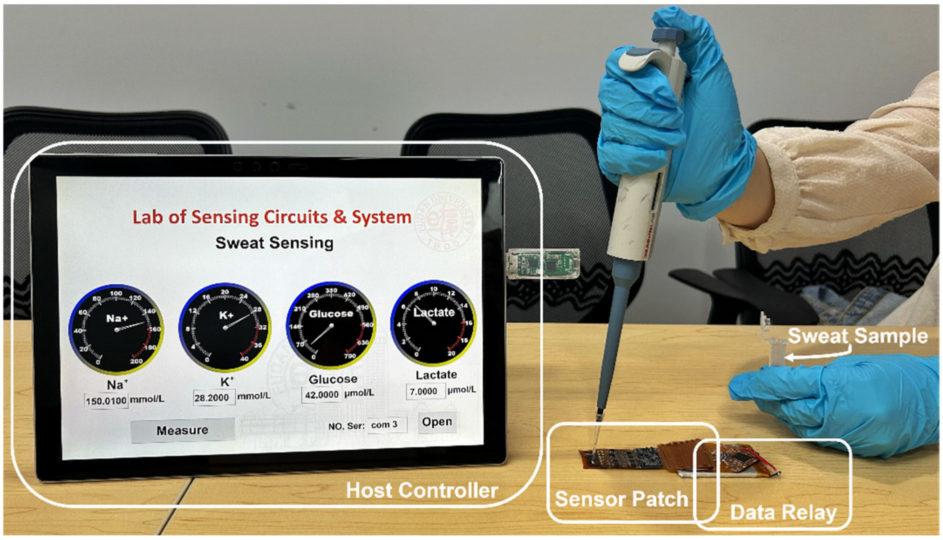

Figure S11: The placement and attachment of the components for the complete system.

Table S1: Key components of the NFC AFE.

Component	Function	Device/Value
D_1	Rectifier	CUS08F30
C_1	Decoupling Capacitor	1μF, 0402
D_2	Zener Diode	MMSZ4686
IC_1	LDO	LP5907-3.3
C_2	Decoupling Capacitor	1μF, 0402
IC_2	Clock Recovery	SN74LVC1G04DRLR
D_3	Demodulator	CUS08F30
R_1	Demodulator	3.6kΩ, 0402
C_3	Demodulator	43pF, 0402
R_2	LPF	7.5kΩ, 0402
C_4	LPF	12pF, 0402
R_3	Modulator	100Ω, 0402
M_1	Modulator	BSS123
